# Necrosis of the Tibia

**Published:** 1855-01

**Authors:** 


### NECROSIS OF THE TIBIA.

Since the case of lithotomy, two others have been operated upon before the class. One of extensive necrosis of the tibia, which dead portion was removed, and the patient is doing well.

Other cases are on the list of applicants for operation, and will shortly come before the class.

To those desiring the benefits of gratuitous operations, if done before the class, we would say that the clinics are on Wednesdays and Saturdays.

